# Bis(*N*,*N*-dimethyl­formamide-κ*O*)bis­(2,4,6-trinitro­phenolato-κ^2^ *O* ^1^,*O* ^2^)copper(II)

**DOI:** 10.1107/S1600536808039846

**Published:** 2008-12-03

**Authors:** Bai-Yu Li, Jun-Feng Tong, Wen-Kui Dong, Jian Yao, Jian-Chao Wu

**Affiliations:** a School of Chemical and Biological Engineering, Lanzhou Jiaotong University, Lanzhou 730070, People’s Republic of China

## Abstract

The mol­ecule of the title complex, [Cu(C_6_H_2_N_3_O_7_)_2_(C_3_H_7_NO)_2_], is disposed about a crystallographic centre of symmetry. The Cu^II^ cation is six-coordinated by two phenolate O atoms and two *ortho*-nitro O atoms of two picrate units and by two carbonyl O atoms from two coordinated dimethyl­formamide mol­ecules, forming a distorted octa­hedral geometry.

## Related literature

For background to 2,4,6-trinitro­phenoxides, see: Arnaud-Neu *et al.* (2005 ▶); Dong *et al.* (1998 ▶, 2007*a* ▶,*b* ▶); Harrowfield *et al.* (1995 ▶, 1998 ▶); Liu *et al.* (2008 ▶); Marchand *et al.* (2003 ▶); Muthamizhchelvan *et al.* (2005 ▶); Song *et al.* (2007 ▶); Talanova *et al.* (1999 ▶); Venkatasubramanian *et al.* (1985 ▶); Wang *et al.* (2003 ▶). 
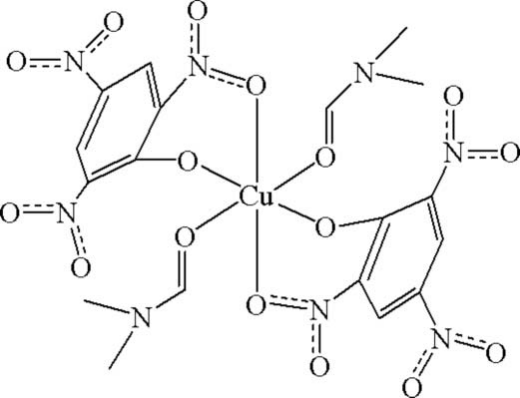


## Experimental

### 

#### Crystal data

[Cu(C_6_H_2_N_3_O_7_)_2_(C_3_H_7_NO)_2_]
*M* *_r_* = 665.94
Triclinic, 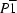

*a* = 8.0620 (10) Å
*b* = 8.3361 (11) Å
*c* = 9.8429 (14) Å
α = 73.945 (1)°
β = 88.796 (2)°
γ = 87.968 (2)°
*V* = 635.25 (15) Å^3^
*Z* = 1
Mo *K*α radiation
μ = 0.96 mm^−1^
*T* = 298 (2) K
0.45 × 0.42 × 0.30 mm

#### Data collection

Siemens SMART 1000 CCD diffractometer
Absorption correction: multi-scan (*SADABS*; Sheldrick, 1996 ▶) *T*_min_ = 0.673, *T*_max_ = 0.762
3320 measured reflections
2198 independent reflections
1973 reflections with *I* > 2σ(*I*)
*R*_int_ = 0.019

#### Refinement

*R*[*F* ^2^ > 2σ(*F* ^2^)] = 0.030
*wR*(*F* ^2^) = 0.078
*S* = 1.08
2198 reflections
199 parameters
H-atom parameters constrained
Δρ_max_ = 0.22 e Å^−3^
Δρ_min_ = −0.36 e Å^−3^

### 

Data collection: *SMART* (Siemens, 1996 ▶); cell refinement: *SAINT* (Siemens, 1996 ▶); data reduction: *SAINT*; program(s) used to solve structure: *SHELXS97* (Sheldrick, 2008 ▶); program(s) used to refine structure: *SHELXL97* (Sheldrick, 2008 ▶); molecular graphics: *SHELXTL* (Sheldrick, 2008 ▶); software used to prepare material for publication: *SHELXTL*.

## Supplementary Material

Crystal structure: contains datablocks global, I. DOI: 10.1107/S1600536808039846/hg2446sup1.cif

Structure factors: contains datablocks I. DOI: 10.1107/S1600536808039846/hg2446Isup2.hkl

Additional supplementary materials:  crystallographic information; 3D view; checkCIF report

## Figures and Tables

**Fig. 1.**
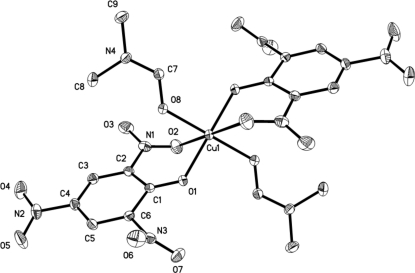
The molecule structure of the title complex with atom numbering scheme [Symmetry codes: -x + 1,-y + 1,-z + 1]. Displacement ellipsoids for non-hydrogen atoms are drawn at the 30% probability level.

## supplementary crystallographic information

### Comment

2,4,6-Trinitrophenoxides, 'picrates', play an important role in the modern coordination chemistry (Dong *et al.*, 1998; Dong *et al.*, 2007*a*). Picrate anion with extraordinary varieties in the binding of complexes, has great potential in building coordination networks (Liu *et al.*, 2008). They can act as the bridging mono- (Arnaud-Neu *et al.*, 2005; Wang *et al.*, 2003), di- (Marchand *et al.*, 2003; Song *et al.*, 2007), tri- (Harrowfield *et al.*, 1995; Dong *et al.*, 1998; Dong *et al.*, 2007*a*), tetra- (Venkatasubramanian *et al.*, 1985) or penta- (Dong *et al.*, 2007*b*; Harrowfield *et al.*, 1998) dentate ligands *via* the phenolic oxygen, *ortho*-nitro oxygen and *para*-nitro oxygen atoms to build coordination networks as well as interlink the one-dimensional or two-dimensional molecules into frameworks *via* the hydrogen bonds (Muthamizhchelvan *et al.*, 2005) or π-π stacking interactions (Talanova *et al.*, 1999). Here, in continuation of our previous studies on synthesis and structural characterization of transition metal complexes with salen-type bisoxime chelating ligands, a single-crystal of unexpected complex, bis(*N*,*N*-dimethylformamide-κ*O*)bis(2,4,6-trinitrophenolato- κ^2^ O,*O*')copper(II), was obtained and structurally characterized by X-ray crystallography.

The crystal structure of the title complex consists of discrete C_18_H_18_CuN_8_O_16_ molecules (Fig. 1), in which all bond lengths are in normal ranges. The two benzene rings in each molecule of the title complex are parallel and the distance between them is 2.115 (2)Å. The central Cu^II^ atom is located on a crystallographic inversion center. The carbonyl oxygens O8, O8^i^ and the phenoxy oxygens O1, O1^i^(symmetry code (i) -x=1, -y+1, -z+1) coordinate to the copper atom to form a distorted square planar structure with Cu1-O2 and Cu1-O8 bond lengths of 1.9226 (15) and 1.9401 (15)Å respectively. The two *ortho*-nitro oxygen atoms (O2 and O2^i^) occupy axial positions with Cu1-O2 = 2.659 (2)Å to give a distorted octahedral coordination geometry around the copper atom.

### Experimental

Copper(II) picrate tetrahydrate and 5,5'-dihydroxy-2,2'-[1,1'-(propane-1,3-diyldioxydinitrilo)diethlidyne]diphenol were synthesized by an analogous method (Dong *et al.*, 2007*a*). A ethyl acetate-*N*,*N*-dimethylformamide mixed solution (2 ml) of 5,5'-dihydroxy-2,2'-[1,1'-(propane-1,3-diyldioxydinitrilo)diethlidyne]diphenol (4.1 mg, 0.01 mmol) was added dropwise to a acetone solution (2 ml) of copper(II) picrate tetrahydrate (6.1 mg, 0.01 mmol) at room temperature. The brilliant yellow solution obtained was placed in n-hexane sphere and allowed to stand at room temperature for about several weeks. Along with diffusion of n-hexane into the mixed solution of the complex, Green block-like single crystals of bis(*N*,*N*-dimethylformamide-κ*O*)bis(2,4,6-trinitrophenolato- κ^2^ O,*O*')copper(II) complex suitable for X-ray crystallographic analysis were obtained. Anal. Calc. for C_18_H_18_CuN_8_O_16_: C, 34,51; H, 3.48; N, 16.10; Cu, 9.13%. Found: C, 34,73; H, 3.51; N, 16.17; Cu, 9.01%.

### Refinement

Non-H atoms were refined anisotropically. H atoms were treated as riding atoms with distances C—H = 0.96 (CH_3_), 0.97 (CH_2_), 0.93 Å (CH), *U*_iso_(H) = 1.2 *U*_eq_(C) and 1.5 *U*_eq_(C).

### Crystal data

**Table:**

[Cu(C_6_H_2_N_3_O_7_)_2_(C_3_H_7_NO)_2_]	*Z* = 1
*M**_r_* = 665.94	*F*(000) = 339
Triclinic, *P*1	*D*_x_ = 1.741 Mg m^−^^3^
Hall symbol: -P 1	Mo *K*α radiation, λ = 0.71073 Å
*a* = 8.062 (1) Å	Cell parameters from 2174 reflections
*b* = 8.3361 (11) Å	θ = 2.5–27.5°
*c* = 9.8429 (14) Å	µ = 0.96 mm^−^^1^
α = 73.945 (1)°	*T* = 298 K
β = 88.796 (2)°	Block-like, green
γ = 87.968 (2)°	0.45 × 0.42 × 0.30 mm
*V* = 635.25 (15) Å^3^	

### Data collection

**Table:**

Siemens SMART 1000 CCD area-detector diffractometer	2198 independent reflections
Radiation source: fine-focus sealed tube	1973 reflections with *I* > 2σ(*I*)
graphite	*R*_int_ = 0.020
φ and ω scans	θ_max_ = 25.0°, θ_min_ = 2.2°
Absorption correction: multi-scan (*SADABS*; Sheldrick, 1996)	*h* = −9→9
*T*_min_ = 0.673, *T*_max_ = 0.762	*k* = −9→6
3320 measured reflections	*l* = −11→11

### Refinement

**Table:**

Refinement on *F*^2^	Secondary atom site location: difference Fourier map
Least-squares matrix: full	Hydrogen site location: inferred from neighbouring sites
*R*[*F*^2^ > 2σ(*F*^2^)] = 0.030	H-atom parameters constrained
*wR*(*F*^2^) = 0.078	*w* = 1/[σ^2^(*F*_o_^2^) + (0.036*P*)^2^ + 0.273*P*] where *P* = (*F*_o_^2^ + 2*F*_c_^2^)/3
*S* = 1.08	(Δ/σ)_max_ < 0.001
2198 reflections	Δρ_max_ = 0.22 e Å^−^^3^
199 parameters	Δρ_min_ = −0.36 e Å^−^^3^
0 restraints	Extinction correction: *SHELXL97* (Sheldrick, 2008), Fc^*^=kFc[1+0.001xFc^2^λ^3^/sin(2θ)]^-1/4^
Primary atom site location: structure-invariant direct methods	Extinction coefficient: 0.141 (6)

### Special details

**Table:**

Geometry. All e.s.d.'s (except the e.s.d. in the dihedral angle between two l.s. planes) are estimated using the full covariance matrix. The cell e.s.d.'s are taken into account individually in the estimation of e.s.d.'s in distances, angles and torsion angles; correlations between e.s.d.'s in cell parameters are only used when they are defined by crystal symmetry. An approximate (isotropic) treatment of cell e.s.d.'s is used for estimating e.s.d.'s involving l.s. planes.
Refinement. Refinement of *F*^2^ against ALL reflections. The weighted *R*-factor *wR* and goodness of fit *S* are based on *F*^2^, conventional *R*-factors *R* are based on *F*, with *F* set to zero for negative *F*^2^. The threshold expression of *F*^2^ > σ(*F*^2^) is used only for calculating *R*-factors(gt) *etc*. and is not relevant to the choice of reflections for refinement. *R*-factors based on *F*^2^ are statistically about twice as large as those based on *F*, and *R*- factors based on ALL data will be even larger.

### Fractional atomic coordinates and isotropic or equivalent isotropic displacement parameters (Å^2^)

**Table:**

	*x*	*y*	*z*	*U*_iso_*/*U*_eq_	
Cu1	0.5000	0.5000	0.5000	0.02554 (17)	
N1	0.5597 (3)	0.5206 (2)	0.1667 (2)	0.0339 (5)	
N2	0.8515 (3)	1.0013 (3)	−0.1186 (2)	0.0407 (5)	
N3	0.5610 (3)	1.0497 (2)	0.3079 (2)	0.0341 (5)	
N4	0.9697 (2)	0.3374 (2)	0.4240 (2)	0.0289 (4)	
O1	0.4863 (2)	0.71913 (19)	0.36927 (16)	0.0332 (4)	
O2	0.4407 (2)	0.4683 (2)	0.2441 (2)	0.0497 (5)	
O3	0.6381 (3)	0.4363 (2)	0.1017 (2)	0.0496 (5)	
O4	0.9214 (3)	0.9088 (3)	−0.1814 (2)	0.0587 (6)	
O5	0.8672 (3)	1.1525 (3)	−0.1527 (2)	0.0639 (6)	
O6	0.6713 (3)	1.1133 (3)	0.3553 (2)	0.0552 (5)	
O7	0.4137 (3)	1.0653 (2)	0.3315 (2)	0.0556 (5)	
O8	0.72371 (18)	0.47226 (19)	0.43167 (16)	0.0306 (4)	
C1	0.5606 (3)	0.7778 (3)	0.2512 (2)	0.0255 (5)	
C2	0.6088 (3)	0.6916 (3)	0.1486 (2)	0.0265 (5)	
C3	0.7030 (3)	0.7633 (3)	0.0293 (2)	0.0301 (5)	
H3	0.7350	0.7018	−0.0331	0.036*	
C4	0.7490 (3)	0.9268 (3)	0.0041 (2)	0.0298 (5)	
C5	0.7014 (3)	1.0223 (3)	0.0951 (2)	0.0287 (5)	
H5	0.7312	1.1331	0.0767	0.034*	
C6	0.6098 (3)	0.9480 (3)	0.2118 (2)	0.0259 (5)	
C7	0.8263 (3)	0.3548 (3)	0.4840 (2)	0.0278 (5)	
H7	0.7989	0.2774	0.5687	0.033*	
C8	1.0134 (3)	0.4484 (4)	0.2868 (3)	0.0434 (6)	
H8A	0.9757	0.4034	0.2134	0.065*	
H8B	1.1317	0.4587	0.2795	0.065*	
H8C	0.9615	0.5564	0.2768	0.065*	
C9	1.0878 (3)	0.2028 (3)	0.4895 (3)	0.0402 (6)	
H9A	1.0476	0.1441	0.5817	0.060*	
H9B	1.1933	0.2489	0.4979	0.060*	
H9C	1.1001	0.1270	0.4320	0.060*	

### Atomic displacement parameters (Å^2^)

**Table:**

	*U*^11^	*U*^22^	*U*^33^	*U*^12^	*U*^13^	*U*^23^
Cu1	0.0244 (2)	0.0238 (2)	0.0255 (2)	−0.00033 (15)	0.00563 (15)	−0.00252 (15)
N1	0.0396 (12)	0.0273 (11)	0.0347 (11)	−0.0003 (9)	−0.0077 (9)	−0.0081 (9)
N2	0.0390 (12)	0.0539 (15)	0.0278 (11)	−0.0100 (11)	0.0067 (9)	−0.0084 (10)
N3	0.0480 (13)	0.0224 (10)	0.0299 (10)	0.0035 (9)	0.0070 (9)	−0.0051 (8)
N4	0.0255 (10)	0.0290 (10)	0.0325 (10)	0.0012 (8)	0.0019 (8)	−0.0097 (8)
O1	0.0358 (9)	0.0260 (8)	0.0316 (9)	0.0026 (7)	0.0142 (7)	0.0011 (7)
O2	0.0463 (11)	0.0393 (10)	0.0610 (12)	−0.0169 (9)	0.0065 (9)	−0.0085 (9)
O3	0.0708 (14)	0.0310 (10)	0.0510 (12)	0.0033 (9)	−0.0010 (10)	−0.0187 (9)
O4	0.0601 (13)	0.0758 (15)	0.0440 (12)	−0.0094 (11)	0.0237 (10)	−0.0236 (11)
O5	0.0878 (17)	0.0495 (13)	0.0479 (12)	−0.0253 (12)	0.0274 (11)	−0.0019 (10)
O6	0.0705 (14)	0.0512 (12)	0.0538 (12)	−0.0025 (11)	−0.0067 (10)	−0.0306 (10)
O7	0.0498 (13)	0.0475 (12)	0.0714 (14)	0.0092 (9)	0.0224 (10)	−0.0225 (10)
O8	0.0249 (8)	0.0303 (9)	0.0318 (9)	0.0019 (7)	0.0065 (6)	−0.0015 (7)
C1	0.0218 (11)	0.0253 (11)	0.0256 (11)	0.0035 (9)	−0.0002 (9)	−0.0013 (9)
C2	0.0273 (12)	0.0235 (11)	0.0274 (11)	0.0003 (9)	−0.0013 (9)	−0.0047 (9)
C3	0.0316 (12)	0.0328 (13)	0.0262 (12)	0.0031 (10)	0.0000 (9)	−0.0088 (9)
C4	0.0286 (12)	0.0348 (13)	0.0226 (11)	−0.0015 (10)	0.0024 (9)	−0.0026 (9)
C5	0.0300 (12)	0.0239 (11)	0.0292 (12)	−0.0041 (9)	0.0008 (9)	−0.0020 (9)
C6	0.0268 (11)	0.0247 (11)	0.0259 (11)	0.0031 (9)	0.0010 (9)	−0.0069 (9)
C7	0.0284 (12)	0.0273 (12)	0.0267 (11)	−0.0027 (9)	0.0025 (9)	−0.0060 (9)
C8	0.0360 (14)	0.0516 (16)	0.0381 (14)	−0.0010 (12)	0.0132 (11)	−0.0059 (12)
C9	0.0323 (13)	0.0369 (14)	0.0517 (16)	0.0065 (11)	−0.0001 (11)	−0.0135 (12)

### Geometric parameters (Å, °)

**Table:**

Cu1—O1^i^	1.9226 (15)	O1—C1	1.275 (3)
Cu1—O1	1.9226 (15)	O8—C7	1.261 (3)
Cu1—O8	1.9401 (15)	C1—C6	1.431 (3)
Cu1—O8^i^	1.9401 (15)	C1—C2	1.432 (3)
Cu1—O2	2.659 (2)	C2—C3	1.385 (3)
N1—O3	1.226 (3)	C3—C4	1.379 (3)
N1—O2	1.228 (3)	C3—H3	0.9300
N1—C2	1.455 (3)	C4—C5	1.393 (3)
N2—O5	1.222 (3)	C5—C6	1.361 (3)
N2—O4	1.230 (3)	C5—H5	0.9300
N2—C4	1.452 (3)	C7—H7	0.9300
N3—O6	1.215 (3)	C8—H8A	0.9600
N3—O7	1.215 (3)	C8—H8B	0.9600
N3—C6	1.474 (3)	C8—H8C	0.9600
N4—C7	1.310 (3)	C9—H9A	0.9600
N4—C8	1.455 (3)	C9—H9B	0.9600
N4—C9	1.459 (3)	C9—H9C	0.9600
			
O1^i^—Cu1—O1	180.0	C3—C2—N1	116.6 (2)
O1^i^—Cu1—O8	90.87 (6)	C1—C2—N1	120.38 (19)
O1—Cu1—O8	89.13 (6)	C4—C3—C2	119.3 (2)
O1^i^—Cu1—O8^i^	89.13 (6)	C4—C3—H3	120.4
O1—Cu1—O8^i^	90.87 (6)	C2—C3—H3	120.4
O8—Cu1—O8^i^	180.000 (1)	C3—C4—C5	121.5 (2)
O1^i^—Cu1—O2	108.49 (7)	C3—C4—N2	119.6 (2)
O1—Cu1—O2	71.51 (7)	C5—C4—N2	118.9 (2)
O8—Cu1—O2	78.81 (6)	C6—C5—C4	117.8 (2)
O8^i^—Cu1—O2	101.19 (6)	C6—C5—H5	121.1
O3—N1—O2	123.1 (2)	C4—C5—H5	121.1
O3—N1—C2	118.1 (2)	C5—C6—C1	125.5 (2)
O2—N1—C2	118.8 (2)	C5—C6—N3	117.3 (2)
O5—N2—O4	123.1 (2)	C1—C6—N3	117.15 (18)
O5—N2—C4	118.4 (2)	O8—C7—N4	122.7 (2)
O4—N2—C4	118.5 (2)	O8—C7—H7	118.7
O6—N3—O7	125.3 (2)	N4—C7—H7	118.7
O6—N3—C6	117.4 (2)	N4—C8—H8A	109.5
O7—N3—C6	117.3 (2)	N4—C8—H8B	109.5
C7—N4—C8	120.7 (2)	H8A—C8—H8B	109.5
C7—N4—C9	121.3 (2)	N4—C8—H8C	109.5
C8—N4—C9	117.95 (19)	H8A—C8—H8C	109.5
C1—O1—Cu1	130.29 (14)	H8B—C8—H8C	109.5
N1—O2—Cu1	108.53 (14)	N4—C9—H9A	109.5
C7—O8—Cu1	126.60 (14)	N4—C9—H9B	109.5
O1—C1—C6	119.4 (2)	H9A—C9—H9B	109.5
O1—C1—C2	127.7 (2)	N4—C9—H9C	109.5
C6—C1—C2	112.79 (19)	H9A—C9—H9C	109.5
C3—C2—C1	123.0 (2)	H9B—C9—H9C	109.5
			
O8—Cu1—O1—C1	27.0 (2)	N1—C2—C3—C4	178.0 (2)
O8^i^—Cu1—O1—C1	−153.0 (2)	C2—C3—C4—C5	−0.7 (3)
O2—Cu1—O1—C1	−51.52 (19)	C2—C3—C4—N2	178.4 (2)
O3—N1—O2—Cu1	123.9 (2)	O5—N2—C4—C3	167.6 (2)
C2—N1—O2—Cu1	−57.3 (2)	O4—N2—C4—C3	−14.0 (3)
O1^i^—Cu1—O2—N1	−115.98 (15)	O5—N2—C4—C5	−13.3 (3)
O1—Cu1—O2—N1	64.02 (15)	O4—N2—C4—C5	165.1 (2)
O8—Cu1—O2—N1	−28.83 (15)	C3—C4—C5—C6	1.1 (3)
O8^i^—Cu1—O2—N1	151.17 (15)	N2—C4—C5—C6	−177.9 (2)
O1^i^—Cu1—O8—C7	−8.81 (18)	C4—C5—C6—C1	1.4 (3)
O1—Cu1—O8—C7	171.19 (18)	C4—C5—C6—N3	179.6 (2)
O2—Cu1—O8—C7	−117.49 (19)	O1—C1—C6—C5	174.3 (2)
Cu1—O1—C1—C6	−144.15 (17)	C2—C1—C6—C5	−3.9 (3)
Cu1—O1—C1—C2	33.8 (3)	O1—C1—C6—N3	−3.9 (3)
O1—C1—C2—C3	−173.8 (2)	C2—C1—C6—N3	177.93 (19)
C6—C1—C2—C3	4.3 (3)	O6—N3—C6—C5	−56.1 (3)
O1—C1—C2—N1	5.9 (3)	O7—N3—C6—C5	122.8 (2)
C6—C1—C2—N1	−176.03 (19)	O6—N3—C6—C1	122.2 (2)
O3—N1—C2—C3	19.5 (3)	O7—N3—C6—C1	−58.9 (3)
O2—N1—C2—C3	−159.3 (2)	Cu1—O8—C7—N4	173.74 (16)
O3—N1—C2—C1	−160.2 (2)	C8—N4—C7—O8	−4.3 (3)
O2—N1—C2—C1	21.0 (3)	C9—N4—C7—O8	178.2 (2)
C1—C2—C3—C4	−2.2 (3)		

Symmetry codes: (i) −*x*+1, −*y*+1, −*z*+1.
